# Compartmental-modelling-based measurement of murine glomerular filtration rate using ^18^F-fluoride PET/CT

**DOI:** 10.1038/s41598-019-47728-x

**Published:** 2019-08-02

**Authors:** Hyo Sang Lee, Yeon-koo Kang, Hyunjong Lee, Jeong Hee Han, Byung Seok Moon, Seok-Soo Byun, Dong-Wan Chae, Keon Wook Kang, Won Woo Lee

**Affiliations:** 1 Department of Nuclear Medicine, Gangneung Asan Hospital, University of Ulsan College of Medicine, Gangneung, Republic of Korea; 2 Department of Molecular Medicine and Biopharmaceutical Sciences, Graduate School of Convergence Science and Technology, Seoul National University, Seoul, Republic of Korea; 3 Department of Nuclear Medicine, Seoul National University Bundang Hospital, Seoul National University College of Medicine, Seongnam-si, Republic of Korea; 4 Department of Urology, Seoul National University Bundang Hospital, Seoul National University College of Medicine, Seongnam-si, Republic of Korea; 5 Department of Internal Medicine, Seoul National University Bundang Hospital, Seoul National University College of Medicine, Seongnam-si, Republic of Korea; 6 Department of Nuclear Medicine, Seoul National University Hospital, Seoul National University College of Medicine, Seoul, Republic of Korea; 7 Cancer Research Institute, Seoul National University, Seoul, Republic of Korea; 8 Institute of Radiation Medicine, Medical Research Centre, Seoul National University, Seoul, Republic of Korea

**Keywords:** Kidney, Translational research

## Abstract

Accurate measurement of glomerular filtration rate (GFR) is essential for optimal decision making in many clinical settings of renal failure. We aimed to show that GFR can be accurately measured using compartmental tracer kinetic analysis of ^18^F-fluoride dynamic PET/CT. Twenty-three male Sprague-Dawley rats of three experimental groups (cyclosporine-administered [n = 8], unilaterally nephrectomized [n = 8], and control [n = 7]) underwent simultaneous ^18^F-fluoride dynamic PET/CT and reference ^51^Cr-EDTA GFR (GFR_CrEDTA_) test at day 0 and post-intervention day 3. ^18^F-fluoride PET GFR (GFR_F-PET_) was calculated by multiplying the influx rate and functional kidney volume in a single-tissue-compartmental kinetic model. Within-test repeatability and between-test agreement were evaluated by intraclass correlation coefficient (ICC) and Bland-Altman analysis. In the control group, repeatability of GFR_F-PET_ was excellent (ICC = 0.9901, repeatability coefficient = 12.5%). GFR_F-PET_ significantly decreased in the renally impaired rats in accordance with respective GFR_CrEDTA_ changes. In the pooled population, GFR_F-PET_ agreed well with GFR_CrEDTA_ with minimal bias (−2.4%) and narrow 95% limits of agreement (−25.0% to 20.1%). These data suggest that the single-compartmental kinetic analysis of ^18^F-fluoride dynamic PET/CT is an accurate method for GFR measurement. Further studies in humans are warranted.

## Introduction

The glomerular filtration rate (GFR) is a widely accepted measure of global renal function, and accurate measurement of GFR is essential for optimal decision making in many clinical settings of renal failure^1^. The GFR has been typically measured as the urinary clearance of an ideal filtration marker such as inulin^2^. Alternatively, plasma clearance of a filtration marker, such as ^51^Cr-ethylenediamine-tetraacetic acid (EDTA), has been advocated for GFR measurement because of its acceptable accuracy without the necessity for tricky urine handling^3^. However, its drawbacks include the requirement for multiple blood samplings and a time-consuming procedure.

Nuclear medicine imaging techniques offer various means of GFR quantitation. Planar renal scintigraphy using ^99m^Tc-diethylenetriamine-pentaacetic acid (DTPA) can provide imaging-based estimation of GFR via Gates’ method^4^. However, the GFR calculated from the Gates’ formula was reported to be less accurate than measured or estimated GFR, probably due to the potential errors in the correction of background and kidney depth, inherent limitations of two-dimensional images^5,6^. Positron emission tomography (PET) enables dynamic 3-dimensional imaging, allowing accurate measurement of input function and tissue concentration of radiotracers, therefore has the potential for quantitative renal imaging^7^. Several proof-of-concept studies produced promising results. ^68^Ga-1,4,7-triaza-cyclononane-1,4,7-triacetic acid (^68^Ga-NOTA) or ^68^Ga-EDTA have been investigated for GFR measurement but the results are yet to be validated^8,9^. To date, there is no accepted methodological standard of PET for GFR measurement.

^18^F-fluoride is an established skeletal PET radiopharmaceutical, but it could also be used for renal imaging because fluoride is not bound to plasma protein and thus is freely filtered through glomeruli^10^. However, fluoride clearance is always lower than GFR due to significant tubular reabsorption^11,12^. Therefore, the previous ^18^F-fluoride dynamic PET/CT study reported a moderate correlation of fluoride clearance with a broad range of renal function parameters; the direct measurement of GFR was beyond the scope^13^.

Compartmental tracer kinetic modelling enables the measurement of rate constants as parameters of important physiological processes *in vivo*. Dynamic PET is suited for this purpose due to its accurate and non-invasive quantification ability. We hypothesized that because the compartmental modelling allows the separate quantification of influx and efflux rates, we might be able to quantify GFR using ^18^F-fluoride influx rate despite the presence of tubular reabsorption. In this study, we showed that GFR could be accurately measured in rats via compartmental modelling of dynamic ^18^F-fluoride PET/CT. Neither urine handling nor blood sampling was necessary in this imaging-based approach. Validity of the compartmental model was independently tested by calculating GFR using dynamic PET/CT scans of ^68^Ga-NOTA.

## Results

### Within-test repeatability

The single-tissue-compartmental model provided excellent goodness-of-fit to the ^18^F-fluoride renal cortical time-activity curve (TAC) (median R^2^ = 0.9674 [inter-quartile range (IQR) = 0.9538–0.9763]). The results of the parameter estimation are summarized in Table 1. The renal cortical volume V_C_ between paired measurements was highly concordant (intraclass correlation coefficient [ICC] = 0.9846 [95% confidence interval (CI) = 0.9802–0.9946], repeatability coefficient = 3.1%), which suggests the reproducibility of the manual drawing of the volumes of interest (VOIs).

**Table 1:** Model parameters.

Group		Number of kidneys	V_C_ (cm^3^)	vB	K_1_ (ml/cm^3^/min)	k_2_ (min^−1^)
Cyclosporine	Baseline	16	1.022 ± 0.074	0.111 ± 0.034	1.109 ± 0.259	0.802 ± 0.179
	Post	16	1.021 ± 0.074	0.125 ± 0.036	0.978 ± 0.244	0.764 ± 0.215
Nephrectomy	Baseline	16	1.150 ± 0.072	0.106 ± 0.027	0.967 ± 0.175	0.783 ± 0.221
	Post	8	1.178 ± 0.057	0.133 ± 0.039	1.043 ± 0.100	0.958 ± 0.024
Control	1st	14	1.067 ± 0.135	0.093 ± 0.026	1.009 ± 0.269	0.787 ± 0.215
	2nd	14	1.065 ± 0.135	0.105 ± 0.029	1.043 ± 0.277	0.820 ± 0.215

The figures are expressed as mean ± standard deviation.

V_C_, renal cortical volume; vB, blood volume fraction; K_1_, influx constant; k_2_, efflux constant.

**Figure 1:**
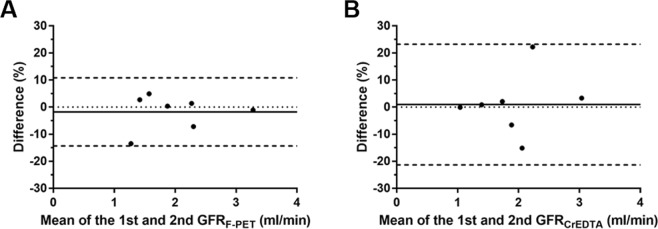
Bland-Altman plots for repeatability of (**A**) GFR_F-PET_ and (**B**) GFR_CrEDTA_. The solid lines represent biases, and the dashed lines represent 95% limits of agreement. Difference (%) = 100 × (GFR_1st_ − GFR_2nd_)/(mean of GFR_1st_ and GFR_2nd_).

The repeatability of ^18^F-fluoride PET GFR (GFR_F-PET_) was excellent (ICC = 0.9901 [95% CI = 0.9501–0.9982], repeatability coefficient = 12.5%), whereas the repeatability of ^51^Cr-EDTA GFR (GFR_CrEDTA_) was slightly lower than that of GFR_F-PET_ (ICC = 0.9372 [95% CI = 0.7155–0.9887], repeatability coefficient = 22.2%; Fig. 1).

### Between-test agreement

GFR_F-PET_ and GFR_CrEDTA_ (Table 2) fell near the reported range of ^51^Cr-EDTA plasma clearance in rats (1.50–3.0 mL/min)^14^. Body surface areas (BSAs) of the rats were estimated as 413 ± 16 cm^2^ (range = 380–455 cm^2^). The BSA-normalized GFR_F-PET_ (range = 41.2–140.2 mL/min/1.73 m^2^) and GFR_CrEDTA_ (range = 44.2–127.6 mL/min/1.73 m^2^) were well-matched with BSA-normalized human GFR.

**Table 2:** GFR in subgroups.

Subgroup	^18^F-fluoride PET GFR (ml/min)			^51^Cr-EDTA GFR (ml/min)		
	Baseline	Post	*P*	Baseline	Post	*P*
Cyclosporine	2.01 ± 0.43	1.73 ± 0.33	0.0113	2.08 ± 0.35	1.82 ± 0.38	0.0300
Nephrectomy	1.98 ± 0.34	1.06 ± 0.08	0.0001	1.97 ± 0.35	1.21 ± 0.07	0.0009
Control	1.98 ± 0.69	2.01 ± 0.69	0.4415	1.93 ± 0.68	1.90 ± 0.62	0.7603

**Figure 2:**
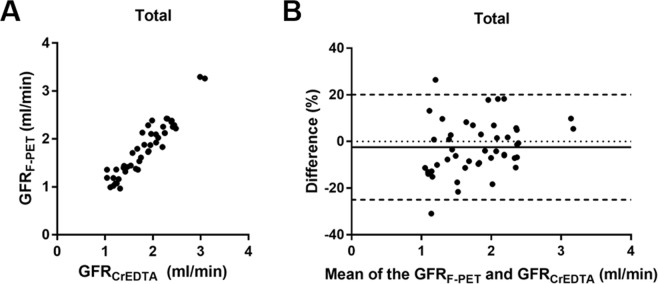
Agreement between GFR_F-PET_ and GFR_CrEDTA_ in the total population (46 measurements). (**A**) The scatterplot. (**B**) The Bland-Altman plot. Difference (%) = 100 × (GFR_F-PET_ − GFR_CrEDTA_)/(mean of GFR_F-PET_ and GFR_CrEDTA_).

The baseline GFR_F-PET_ and GFR_CrEDTA_ were not significantly different among the experimental groups (*P* = 0.830 and 0.686, respectively; Table 2). After cyclosporine intake or nephrectomy, GFR_F-PET_ and GFR_CrEDTA_ were significantly decreased (Supplementary Fig. 1), whereas in the control group, there was no such decrease (Supplementary Fig. 2). In each of the three groups, GFR_F-PET_ and GFR_CrEDTA_ were in good agreement (Supplementary Fig. 3). In the pooled population (46 measurements), GFR_F-PET_ agreed well with GFR_CrEDTA_ (ICC = 0.937 [95% CI = 0.889–0.965]), with minimal bias (−2.4% [relative difference]; −0.027 ml/min [absolute difference]) and narrow 95% limits of agreement (LOA) (−25.0% to 20.1% [relative difference]; −0.401 to 0.347 ml/min [absolute difference]) (Fig. 2, Supplementary Fig. 4). P_30_ and P_10_ (see Statistics in the Methods section) were 97.8% (45/46) and 60.9% (28/46), respectively. The accuracy statistics of the GFR_F-PET_ were summarized in the Table 3.

**Table 3:** Accuracy statistics.

Group	ICC	95% CI for ICC	Relative difference (%)		Absolute difference (ml/min)		P_30_	P_10_
			Bias	LOA	Bias	LOA		
Total	0.937	0.889–0.965	−2.4	−25.0 to 20.1	−0.027	−0.401 to 0.347	97.8 (45/46)	60.9 (28/46)
**Subgroup**								
Cys	0.898	0.740–0.963	−4.5	−22.9 to 13.8	−0.080	−0.396 to 0.236	100 (16/16)	81.3 (13/16)
Nx	0.939	0.839–0.978	−6.5	−28.5 to 15.5	−0.073	−0.395 to 0.248	93.8 (15/16)	50.0 (8/16)
Control	0.941	0.833–0.981	4.6	−17.9 to 27.2	0.086	−0.329 to 0.502	100 (14/14)	57.1 (8/14)

ICC, intraclass correlation coefficient between ^18^F-fluoride PET GFR and ^51^Cr-EDTA GFR; CI, confidence interval; LOA, limits of agreement; Cys, cyclosporine; Nx, nephrectomy.

GFR_F-PET-15min_ showed almost perfect agreement with GFR_F-PET_ (ICC = 0.998 [95% CI = 0.997–0.999], bias = 0.1%, and 95% LOA = −3.3% to 3.5%; Supplementary Fig. 5), which suggests that the two could be used interchangeably and therefore that imaging time could be shortened to 15 min without loss of accuracy.

### Dynamic ^68^Ga-NOTA PET/CT

Overall, ^68^Ga-NOTA showed poorer goodness-of-fit (median R^2^ = 0.5223 [IQR = 0.2295–0.6528] for the 20 kidneys) than did ^18^F-fluoride. The discrepancy between the model curve and kidney TAC was particularly large at later time points (>about 15–20 min). The goodness-of-fit was improved when only the first 15 min of data was used for fitting (median R^2^ = 0.8557 [IQR = 0.8238–0.9001]). Thus, we used ^68^Ga-NOTA PET GFR using first 15 min of data (GFR_NOTA-PET-15min_) for the subsequent analysis.

Because ^68^Ga-NOTA GFR calculation using whole-blood input function produced significant bias, conversion to plasma input function was essential (Supplementary Fig. 6A). After conversion using measured haematocrit, GFR_NOTA-PET-15min_ showed a good agreement with GFR_CrEDTA_ (ICC = 0.9664 [95% CI = 0.8787–0.9914]) with minimal bias (−2.4%) and narrow 95% LOA (−25.9% to 21.1%; Supplementary Fig. 6B). GFR_NOTA-PET-15min_ using a fixed haematocrit of 0.45 showed far wider LOA (−46.8% to 55.5%) than those using measured haematocrit (Supplementary Fig. 6C).

## Discussion

In this study, we developed a compartmental tracer kinetic model for PET-based GFR measurement and applied it to ^18^F-fluoride, which is not a GFR tracer under the conventional concept of urinary or plasma clearance measurement. According to the model, the influx rate K_1_ can be considered as GFR per unit extravascular renal cortical volume for any tracer that is freely filtered through glomeruli but does not undergo tubular secretion. Previous reports suggests that ^18^F-fluoride has such properties^11,12^. GFR_F-PET_ was in good agreement with gold-standard GFR_CrEDTA_ in conditions of nephrotoxic drug use and post-nephrectomy with minimal bias and narrow LOA. P_30_ and P_10_ were 97.8% and 60.9%, respectively, which suggests that GFR_F-PET_ possesses sufficient accuracy (P_30_ > 80% and P_10_ > 50%) compared with other GFR markers such as iohexol, iothalamate and DTPA^15,16^. Furthermore, the accuracy of GFR_F-PET_ was preserved with a reduction in imaging time to 15 min, which bears practical importance.

Good within-test repeatability is a prerequisite for assessing between-test agreement^17^. The repeatability of GFR_F-PET_ was excellent with repeatability coefficient (half-width of the LOA) of 12.6%. GFR_CrEDTA_ measured in this study showed slightly poorer repeatability coefficient of 22.2%, which is somewhat large compared to the reproducibility figures previously reported in humans (7.4–9.0%)^18^. This might have been caused by technical difficulties of the small animal experiment. We speculate that the agreement between GFR_F-PET_ and GFR_CrEDTA_ might be even better in humans, considering the expected increase in the precision of GFR_CrEDTA_.

To our knowledge, approaches of measuring GFR by using a compartmental rate constant have not been attempted in the field of nuclear medicine. In contrast, various types of compartmental modelling approach have been employed in magnetic resonance imaging (MRI) or CT studies. However, a critical literature review suggested that these MRI- or CT-based methodologies are not adequately accurate to be used as routine clinical or research tools^19^. Among the MRI-based methods, the cortical compartment model proposed by Annet *et al*. is similar to ours^20^. The differences are that Annet’s method used two-dimensional regions of interest (ROIs) and abdominal aortic input function and that the dispersion and time delay from aorta to renal vasculature were accounted for. Many MRI-based methods use two-dimensional single-slice ROIs for better temporal resolution, and this acts as a limitation because a single slice or a slab cannot be representative of a whole kidney^20–22^. In this respect, the inherent 3-dimensional capability of PET is an advantage. The use of dispersion- and time-delay-corrected aortic input curves might be a merit of Annet’s method in their rabbit experiment. However, we do not think that the non-correction for dispersion and time-delay caused any significant biases in our rat experiments because of smaller animal size. If this PET/CT analysis is implemented in humans, a proper selection of site for arterial input function measurement may become an important issue.

There may be a concern about the spill-out from the renal pelvic radioactivity into the renal cortical ROIs, considering small size of the rat kidneys. However, the scatter from the renal pelvic radioactivity turned out to be negligible compared with the renal cortical uptake. No significant amount of spill-out activity from the renal pelvis reached the renal cortical ROIs because the renal cortex and renal pelvis are intervened by the renal medulla and because the spatial resolution in terms of full-width half-maximum of the micro PET system used in our study was 0.7 mm that was much smaller than the thickness of the renal medulla (more than 3 mm).

We conducted another set of experiments using ^68^Ga-NOTA. The results also showed good agreement with GFR_CrEDTA_ (Supplementary Fig. 6B). However, the goodness-of-fit to the ^68^Ga-NOTA data was not as good as that for ^18^F-fluoride. The cause of the poor fit is unclear. We speculate that the urination process might not follow first-order (exponential) kinetics and therefore that the process might not be appropriately described by an exponential rate constant k_u_. For ^68^Ga-NOTA, the rate constant k_2_ (=k_u_ + k_reabs_) becomes k_u_ because k_reabs_ = 0, and according to the above speculation, k_2_ also becomes an inappropriately modelled parameter. This could hamper the validity of the model equations. In contrast, ^18^F-fluoride is reabsorbed through the lipid bilayer of tubular cells via passive diffusion^23^, and passive diffusion follows first-order kinetics. The reabsorption of fluoride is approximately 60% of glomerular filtrate, but it could increase up to 90%^11,12^. This implies that k_reabs_ comprises a major portion of the efflux constant k_2_, causing the efflux process to roughly follow first-order kinetics. Therefore, the model fit becomes better for ^18^F-fluoride, which would be a paradoxical advantage of nonzero reabsorption.

Measurement of haematocrit was essential for the calculation of ^68^Ga-NOTA plasma input function because the fixed plasma fraction produced imprecise GFR (Supplementary Fig. 6C). In contrast, a fixed plasma fraction of 1.23 produced accurate GFR for ^18^F-fluoride. It is likely that the plasma fraction of ^18^F-fluoride remained relatively stable irrespective of haematocrit because ^18^F-fluoride permeates into the RBC^24^, whereas the plasma fraction of ^68^Ga-NOTA is more affected by haematocrit because ^68^Ga-NOTA cannot enter in the RBC^8^. The high accuracy of GFR_F-PET_ under a fixed plasma fraction is an advantage because haematocrit need not be measured, eliminating the need for blood sampling.

Given the high accuracy of the GFR measurement using dynamic ^18^F-fluoride PET, translational application to humans may be promising for appropriate indications. Using the expensive PET technology for GFR measurement could only be justified in clinical situations where accurate measurement of GFR is critically necessary. Such situations might include nephron-sparing surgery for malignant lesions in patients with marginal renal function, determination of overall and split renal function before abdominal radiotherapy, and monitoring of renal function during nephrotoxic drug use^9,25^.

The present study has limitations. First, the range of the measured GFR was not sufficiently wide. The normalized GFR_F-PET_ measured in this study fell within 41.2–140.2 mL/min/1.73 m^2^ BSA. Further validation is needed for low GFR values because chronic kidney disease stage grades 4 and 5 (GFR <30 mL/min/1.73 m^2^) were not included in the tested range^26^. Second, manual drawing of ROIs is too laborious for future clinical application. Automatic segmentation of renal cortex might have to be implemented.

In conclusion, dynamic ^18^F-fluoride PET/CT in conjunction with a single-compartmental modelling approach holds promise as a reliable and accurate method for GFR measurement. The difficulties in urine handling and blood sampling in the measurement of conventional urinary and plasma clearance of ideal filtration markers may be overcome by pure image-based analysis. A quick assessment of GFR (within 15 min) is another practical advantage of this approach. Further studies in humans are warranted.

## Materials and Methods

### Tracer kinetic modelling

The compartmental tracer kinetic modelling is a mathematical framework that originated from the field of pharmacokinetics and is a commonly used model for analysing PET data^27^. In the modelling, it is assumed that there are physiologically separate pools, or compartments, of a tracer substance^27^. Each compartment has its own influx and efflux rate constants, and the model fitting procedure allows to quantify them. We devised a compartmental tracer kinetic model in which the rate constant of a certain compartment could be interpreted as GFR.

**Figure 3:**
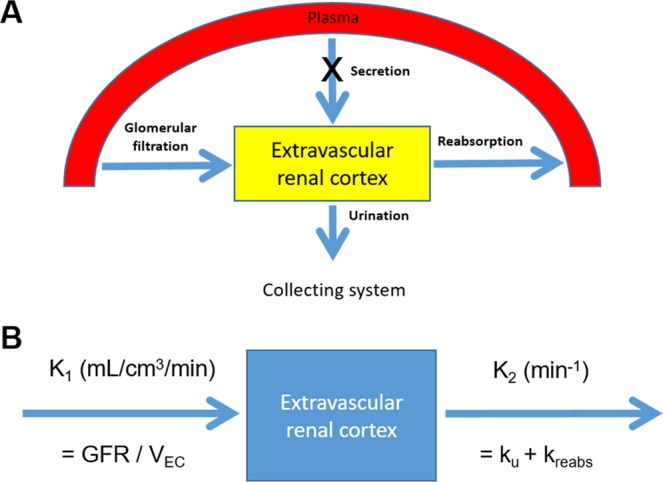
Study concept. (**A**) A schematic diagram of the single-tissue-compartmental model. (**B**) Rate constants in the model.

In the model, extravascular renal cortex (EVRC), which contains Bowman’s capsule, the renal tubule, and the interstitium, serves as a functional kidney volume. A tracer enters the EVRC via glomerular filtration and tubular secretion and moves out via reabsorption and urinary outflow (Fig. 3A). The rate of change in the tracer amount within the EVRC can be described by the following equation:

1 $$\begin{array}{rcl}\frac{d{A}_{EC}(t)}{dt} & = & GFR\times {C}_{P}(t)+{k}_{secr}\times {C}_{P}(t)-{k}_{u}\times {A}_{EC}(t)-{k}_{reabs}\times {A}_{EC}(t)\\  & = & (GFR+{k}_{secr})\times {C}_{P}(t)-({k}_{u}+{k}_{reabs})\times {A}_{EC}(t)\end{array}$$

where A_EC_(t) = tracer amount within EVRC, C_P_(t) = tracer concentration in plasma, k_secr_ = rate constant of tubular secretion, k_u_ = rate constant of tracer loss due to urinary outflow from the cortex, and k_reabs_ = rate constant of tubular reabsorption.

Because no tubular secretion occurs for the ^18^F-fluoride^11,12^, k_secr_ = 0 (Fig. 3A), the Equation (1) becomes as follows:

2 $$\frac{d{A}_{EC}(t)}{dt}=GFR\times {C}_{P}(t)-({k}_{u}+{k}_{reabs})\times {A}_{EC}(t)$$

Dividing the equation by EVRC volume V_EC_ = V_C_ × (1 − vB) gives

3 $$\begin{array}{c}\frac{d}{dt}({A}_{EC}(t)/{V}_{EC})=\frac{GFR}{{V}_{EC}}\times {C}_{P}(t)-({k}_{u}+{k}_{reabs})\times \frac{{A}_{EC}(t)}{{V}_{EC}}\\ \frac{d{C}_{EC}(t)}{dt}={K}_{1}\times {C}_{P}(t)-{k}_{2}\times {C}_{EC}(t)\end{array}$$

where V_C_ = renal cortical volume, vB = vascular volume fraction, C_EC_(t) = tracer concentration within the EVRC, K_1_ = GFR/V_EC_ and k_2_ = k_u_ + k_reabs_ (Fig. 3B).

The solution to Equation (3) can be expressed as follows:

4 $${C}_{EC}(t)={K}_{1}\times {\int }_{0}^{t}{C}_{P}(\tau )\,{e}^{-{k}_{2}(t-\tau )}d\tau ={K}_{1}\times {C}_{P}(t)\otimes {e}^{-{k}_{2}t}$$

where ⊗ = convolution integral.

The model function C_model_(t) can be expressed as a superposition of C_EC_(t) and C_P_(t) according to their respective volume fractions in the kidney:

$${C}_{model}(t)={C}_{EC}(t)\times (1-vB)+{C}_{P}(t)\times vB$$

The C_model_(t) is fitted to the renal cortical TAC with K_1_, k_2_, and vB as fitting parameters. Single-kidney GFR is obtained by multiplying K_1_ and V_C_ × (1 − vB), and total GFR is the sum of the GFR values of both kidneys.

We applied the above model to ^18^F-fluoride dynamic PET/CT to measure the GFR and compared the values with gold-standard ^51^Cr-EDTA GFR. Additionally, we tested the model using ^68^Ga-NOTA. ^68^Ga-NOTA was recently reported as a promising GFR tracer with no tubular reabsorption and secretion, and minimal binding to RBCs and serum protein^8^.

### Radiopharmaceutical preparation

^18^F-fluoride was produced by proton irradiation to the H_2_^18^O target using an in-house cyclotron (KOTRON-13, Samyoung Unitech). ^68^Ga-NOTA was produced by labelling NOTA (ChemaTech) with ^68^Ga eluted from a ^68^Ge/^68^Ga generator (IGG100; Eckert & Ziegler) as previously described^8^.

### Protocol of ^18^F-fluoride dynamic PET/CT Imaging and the ^51^Cr-EDTA Test

Imaging was performed from the thorax to the abdomen in the prone position on a dedicated small-animal PET/CT scanner (NanoScan micro PET/CT 122S; Mediso) under general anaesthesia through isoflurane inhalation (2–3% in 2–5 L/min of oxygen). In each PET/CT imaging sessions, ^18^F-fluoride (3.7 MBq/100 g rat weight in 200 μL solution) and ^51^Cr-EDTA (GE Healthcare; 0.19 MBq in 500 μL solution) were simultaneously injected via the tail vein after the acquisition of the contrast-enhanced CT scan. Immediately following the injection of the radiopharmaceuticals, dynamic ^18^F-fluoride PET images were obtained in the list mode for 60 min with varying frame durations (5 s × 6, 10 s × 3, 15 s × 4, 30 s × 16, 60 s × 20, and 300 s × 6) (please see the Supplementary Methods for PET/CT parameters for acquisition and reconstruction).

**Figure 4:**
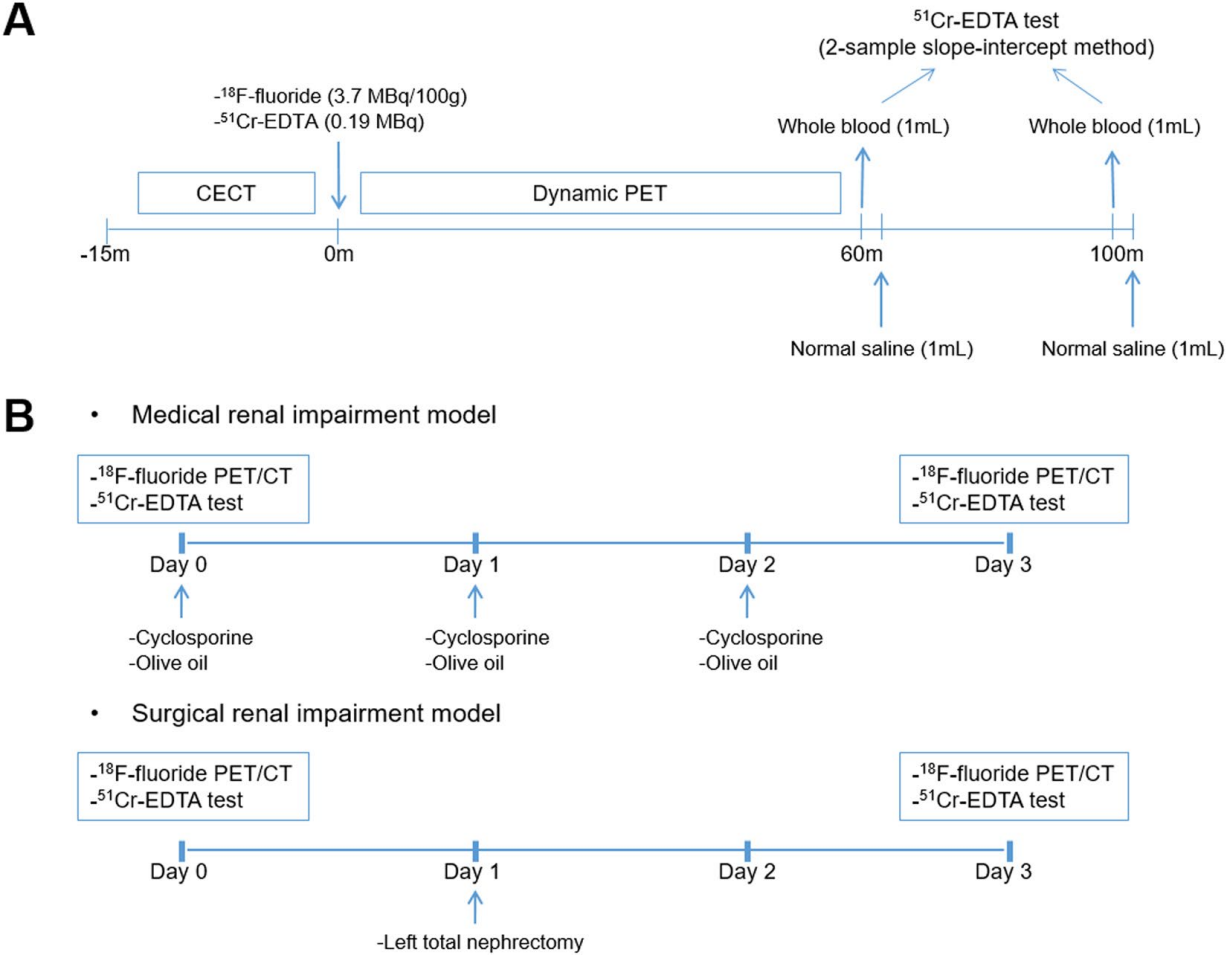
Study design. (**A**) ^18^F-fluoride dynamic PET/CT imaging and ^51^Cr-EDTA test protocol. CECT = contrast-enhanced computed tomography. (**B**) Animal experiment protocol.

After the dynamic PET acquisition, at 60 and 100 min post ^51^Cr-EDTA injection, 1 mL of blood was withdrawn via tail-tip cutting (Fig. 4A). Following each blood withdrawal, 1 mL of saline was flushed to replenish the volume. Plasma samples obtained by centrifugation (3,000 rpm for 8 min) were divided into two aliquots for duplication, and the radioactivity of the plasma aliquots was measured for 20 min using a well counter (Wizard 1480, Perkin Elmer) 24 h after the blood withdrawal to ensure full decay of the PET radiopharmaceuticals. The plasma clearance of ^51^Cr-EDTA was calculated from the mean values of the duplicate counts after background correction using the two-sample slope-intercept method^28^. The slope-intercept plasma clearance was corrected for neglecting the fast exponential in the bi-exponential plasma curve, generating the GFR_CrEDTA_ (please see the Supplementary Methods for details)^29^.

### Animal experiment protocol

For the ^18^F-fluoride PET/CT experiment, 23 male Sprague-Dawley rats (age: 8 weeks; weight: 280 ± 12 g) were used. The rats were divided into three experimental groups. Eight rats were administered with cyclosporine (Sandimmun INJ, Novartis) 30 mg/kg orally from day 0 to 2 to induce renal impairment medically. Another eight rats underwent left total nephrectomy at day 1 to form a surgical renal impairment group. The remaining seven rats were fed 1 mL/day olive oil from day 0 to 2 and served as controls. Each rat underwent two ^18^F-fluoride PET/CT imaging sessions at an interval of 3 days, at baseline (day 0) and after the renal impairment or control procedures (day 3) (Fig. 4B).

For the ^68^Ga-NOTA PET/CT experiment, 10 male naïve Sprague-Dawley rats (334 ± 52 g) underwent dynamic PET/CT and a ^51^Cr-EDTA test. The experimental protocol was the same for the ^68^Ga-NOTA experiment, except for the haematocrit measurement (please see Supplementary Methods) and ^68^Ga-NOTA (3.7 MBq/100 g rat weight) injection.

### Image analysis

**Figure 5:**
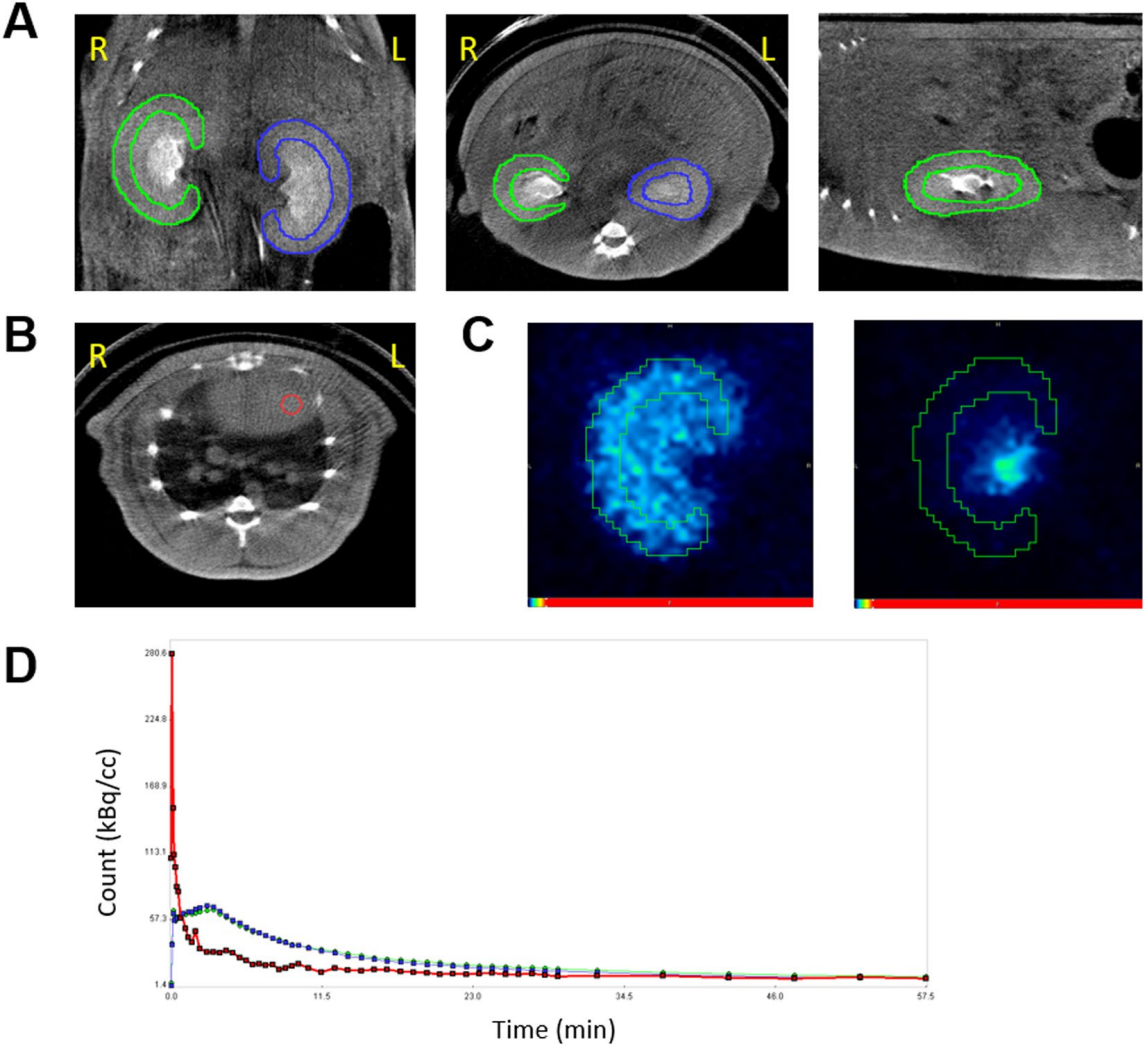
How to analyze the ^18^F-fluoride dynamic PET/CT. (**A**) Renal cortical regions of interest. (**B**) The left ventricular volume of interest. (**C**) ^18^F-fluoride PET images in the renal uptake phase (2.5 to 3 min post-injection; left panel) and excretory phase (25 to 26 min post-injection; right panel). (**D**) Time-activity curves of the right kidney (green), left kidney (blue), and left ventricle (red). R = right, L = left.

We performed PET/CT data analysis and tracer kinetic modelling using PMOD software (version 3.8; PMOD Technologies). ROIs were manually drawn over the renal cortices on the coronal CT images (Fig. 5A), and the ROIs over the same kidney were integrated to form a VOI. A 3-mm-diameter spherical VOI was placed in the left ventricular cavity to obtain whole-blood input function (Fig. 5B). The ROIs was overlaid on the co-registered dynamic PET images to obtain renal cortical TACs (Fig. 5C). In order to convert whole-blood input function to plasma input function, we adopted a fixed plasma fraction of 1.23 for ^18^F-fluoride^30^ because it permeates into RBCs with its intracellular concentration stable with about half in plasma^31,32^. In contrast, we adopted a plasma fraction of 1/(1–hematocrit) for ^68^Ga-NOTA because it does not distribute into RBCs^8^. To test whether the measurement of haematocrit is mandatory for the calculation of ^68^Ga-NOTA plasma input function, we calculated another set of plasma input functions by assuming a fixed haematocrit of 0.45.

The single-tissue-compartmental model curve using the plasma input function was fitted to the renal cortical TACs to obtain GFR_F-PET_ and ^68^Ga-NOTA PET GFR (GFR_NOTA-PET_) (Fig. 5D). Additionally, we calculated PET GFR only using the first 15 min of data (GFR_F-PET-15min_ and GFR_NOTA-PET-15min_) to test the feasibility of reducing imaging time.

### Statistics

The goodness-of-fit of the model was assessed using the coefficient of determination (R^2^). We used the control group data to test for repeatability. Within-test repeatability and between-test agreement were assessed by means of the ICC and the Bland-Altman analysis^17,33^. Accuracy of GFR_F-PET_ was expressed by P_30_ and P_10_, which are defined as the percentages of the measurements that lie within the ±30% and ±10% ranges from reference GFR_CrEDTA_, respectively^15,16^. The paired-samples *t*-test was performed to analyse the difference between paired observations. The Kruskal-Wallis test was performed for group comparisons. Two-sided *P* < 0.05 was considered as significant. All statistical tests were performed using MedCalc statistical software (version 18.5; MedCalc Software bvba).

### Study approval

The rats were cared for in a facility accredited by the Association for Assessment and Accreditation of Laboratory Animal Care International. The study protocol was approved by the Institutional Animal Care and Use Committee of Seoul National University Bundang Hospital (IACUC No. BA1705-223/041-01). All experiments were performed in accordance with relevant guidelines and regulations.

## Supplementary information

Supplementary materials

## Data Availability

The datasets generated during and/or analysed during the current study are available from the corresponding author on reasonable request.
