# Supplementary material for: Compartmental-modelling-based measurement of murine glomerular filtration rate using 18F-fluoride PET/CT
Source: Sci Rep. 2019 Aug 2;9:11269. doi: 10.1038/s41598-019-47728-x (PMC6677809; doi:10.1038/s41598-019-47728-x)
Supplement: Supplementary file 1 — Supplementary materials [file 41598_2019_47728_MOESM1_ESM.docx]

**Compartmental-modelling-based measurement of murine glomerular filtration rate using ^18^F-fluoride** **PET/CT**

Hyo Sang Lee^1^, Yeon-koo Kang^2^, Hyunjong Lee^2^, Jeong Hee Han^3^, Byung Seok Moon^3^, Seok-Soo Byun^4^, Dong-Wan Chae^5^, Keon Wook Kang^6,7^, and *Won Woo Lee^3,8^

^1^Department of Nuclear Medicine, Gangneung Asan Hospital, University of Ulsan College of Medicine, Gangneung, Republic of Korea

^2^Department of Molecular Medicine and Biopharmaceutical Sciences, Graduate School of Convergence Science and Technology, Seoul National University, Seoul, Republic of Korea

Departments of ^3^Nuclear Medicine, ^4^Urology and ^5^Internal Medicine, Seoul National University Bundang Hospital, Seoul National University College of Medicine, Seongnam-si, Republic of Korea

^6^Department of Nuclear Medicine, Seoul National University Hospital, Seoul National University College of Medicine, Seoul, Republic of Korea

^7^Cancer Research Institute, Seoul National University, Seoul, Republic of Korea

^8^Institute of Radiation Medicine, Medical Research Centre, Seoul National University, Seoul, Republic of Korea

Hyo Sang Lee and Yeon-koo Kang equally contributed to the work.

**Corresponding author**: Won Woo Lee, MD, PhD

Department of Nuclear Medicine, Seoul National University Bundang Hospital, Seoul National University College of Medicine. 82 Gumi-ro 173 Beon-gil, Bundang-gu, Seongnam-si, Gyeonggi-do 13620, Republic of Korea

Phone: 82-31-787-7672; Fax: 82-31-787-4018; E-mail: wwlee@snu.ac.kr

**Supplementary Methods**

**Acquisition and reconstruction parameters of ^18^F-fluoride PET/CT**

The contrast-enhanced CT scan (55 kVp, 145 μA, and 1,100 ms exposure time) was first acquired for 15 min before injection of ^18^F-fluoride and ^51^Cr-EDTA. During the CT, an iodinated contrast agent (Ultravist370, Bayer; 3-mL infusion) was continuously infused over 9 min using an auto-injector (NanoJet, Revodix). The CT scan was reconstructed using Butterworth filter, 332×332×473 image matrix and 211-μm isotropic voxel size. PET was reconstructed using an axial field of view of 94.7 mm (1 bed), iterative 3-dimensional expectation maximization algorithm, 110×110×234 image matrix and 0.4-mm isotropic voxel size. PET attenuation correction was performed using the CT attenuation map ^1,2^.

**Correction for the single-exponential approximation and calculation of gold standard ^51^Cr-EDTA GFR**

Fleming et al. suggested a correction method for missed area-under-curve of bi-exponential plasma concentration curve. The equation for corrected GFR (GFR_corr_) was based on body surface area (BSA)-normalized GFR (GFR_BSA_) ^3^.

GFR_BSA_ (mL/min/1.73 m^2^) = GFR_SI_ × 17300/(BSA in cm^2^),

where GFR_SI_ = GFR calculated from the slope-intercept method.

Corrected GFR (GFR_corr_) = GFR_BSA_/(1 + 0.0017 × GFR_BSA_)

The equation for BSA in rats was derived using an updated Meeh’s constant of 9.83 ^4^:

Rat BSA in cm^2^ = 9.83 × (weight in g)^2/3^

The final gold standard GFR for ^51^Cr-EDTA GFR (GFR_CrEDTA_) was calculated by inverting the BSA normalization as following:

GFR_CrEDTA_ (mL/min) = GFR_corr_ (mL/min/1.73m^2^) × (BSA in cm^2^)/17300

**Haematocrit measurement in rats**

After ^18^F-fluoride PET/CT imaging acquisition for 60 min, EDTA-anticoagulated whole blood samples were collected by tail tip cutting of the experiment rats. Haematocrit of collected samples was determined using an automatic blood corpuscle analyser (HEMAVET 950; Drew Scientific, FL, USA) within 60 min from blood sampling.

**References**

1. Han, J. H., Lim, S. Y., Lee, M. S. & Lee, W. W. Sodium [18F]Fluoride PET/CT in Myocardial Infarction. *Mol. Imaging Biol.* **17,** 214–221 (2015).

2. Choi, H. *et al.* Imaging of myocardial ischemia-reperfusion injury using sodium [18F]Fluoride positron emission tomography/computed tomography in rats and humans. *Mol. Imaging* **16,** 1–9 (2017).

3. Fleming, J. S. An improved equation for correcting slope-intercept measurements of glomerular filtration rate for the single exponential approximation. *Nucl. Med. Commun.* **28,** 315–320 (2007).

4. Gouma, E. *et al.* A simple procedure for estimation of total body surface area and determination of a new value of Meeh’s constant in rats. *Lab. Anim.* **46,** 40–45 (2012).

**Supplementary figures and figure legends**


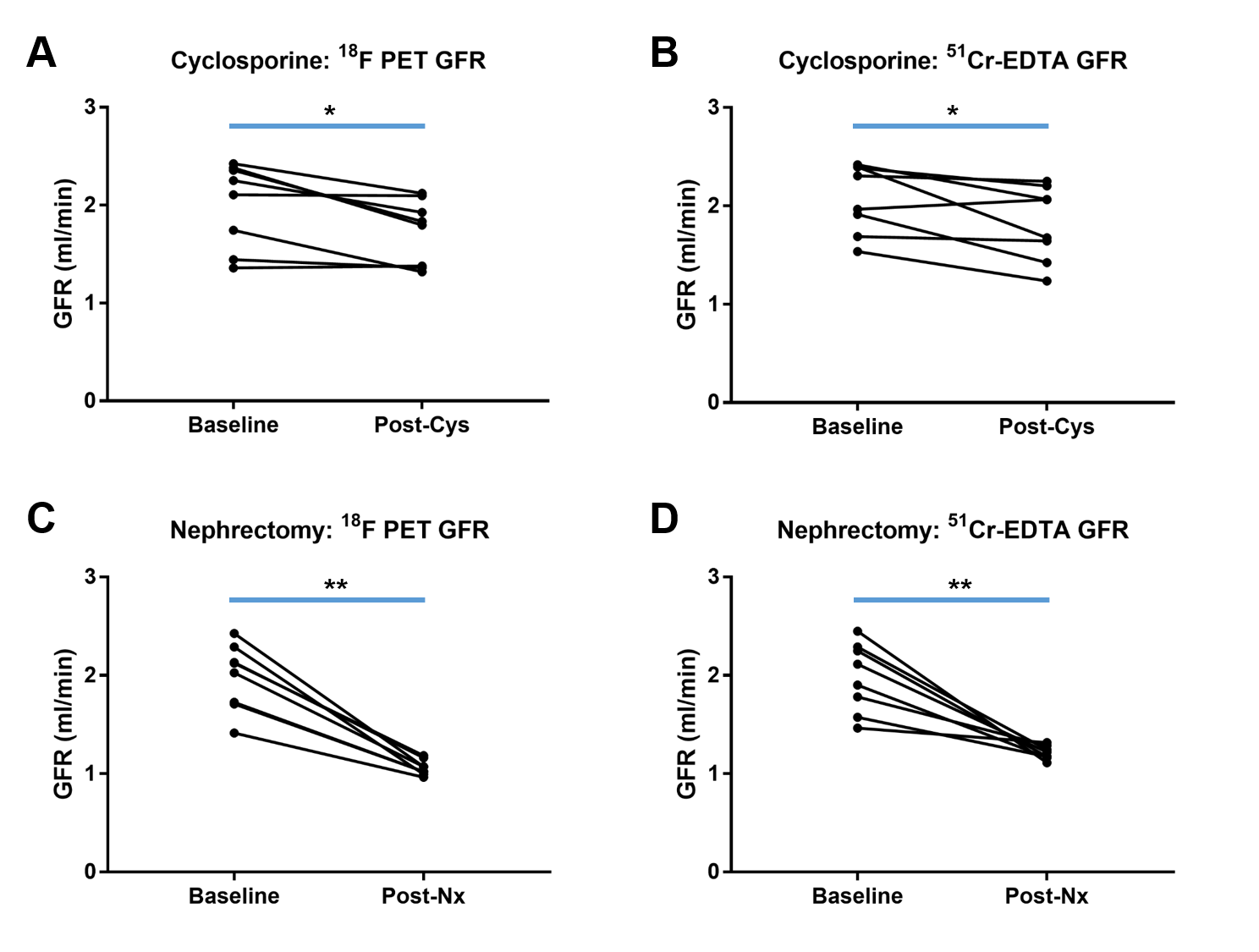


**Supplementary Figure 1.** GFR change after renal function impairment procedures. GFR_F-PET_ and GFR_CrEDTA_ were significantly decreased (A, B) after cyclosporine intake or (C, D) unilateral nephrectomy. *: *P* < 0.05, **: *P* < 0.01.


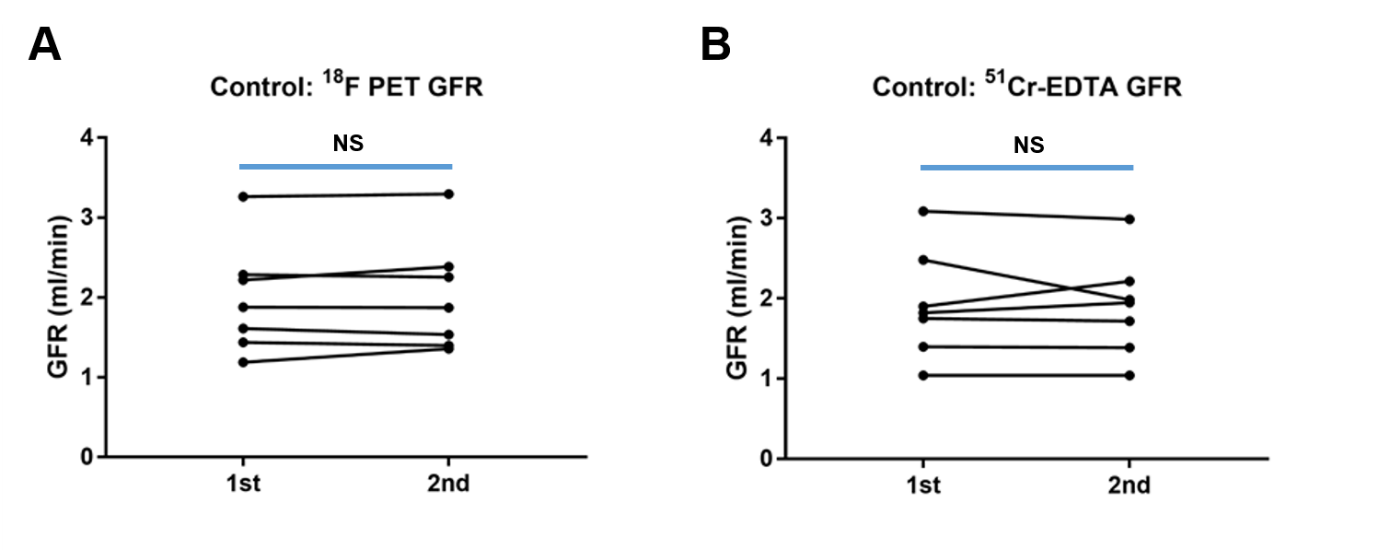


**Supplementary Figure 2.** GFR change in control rats. (A) GFR_F-PET_ and (B) GFR_CrEDTA_. NS: non-significant.


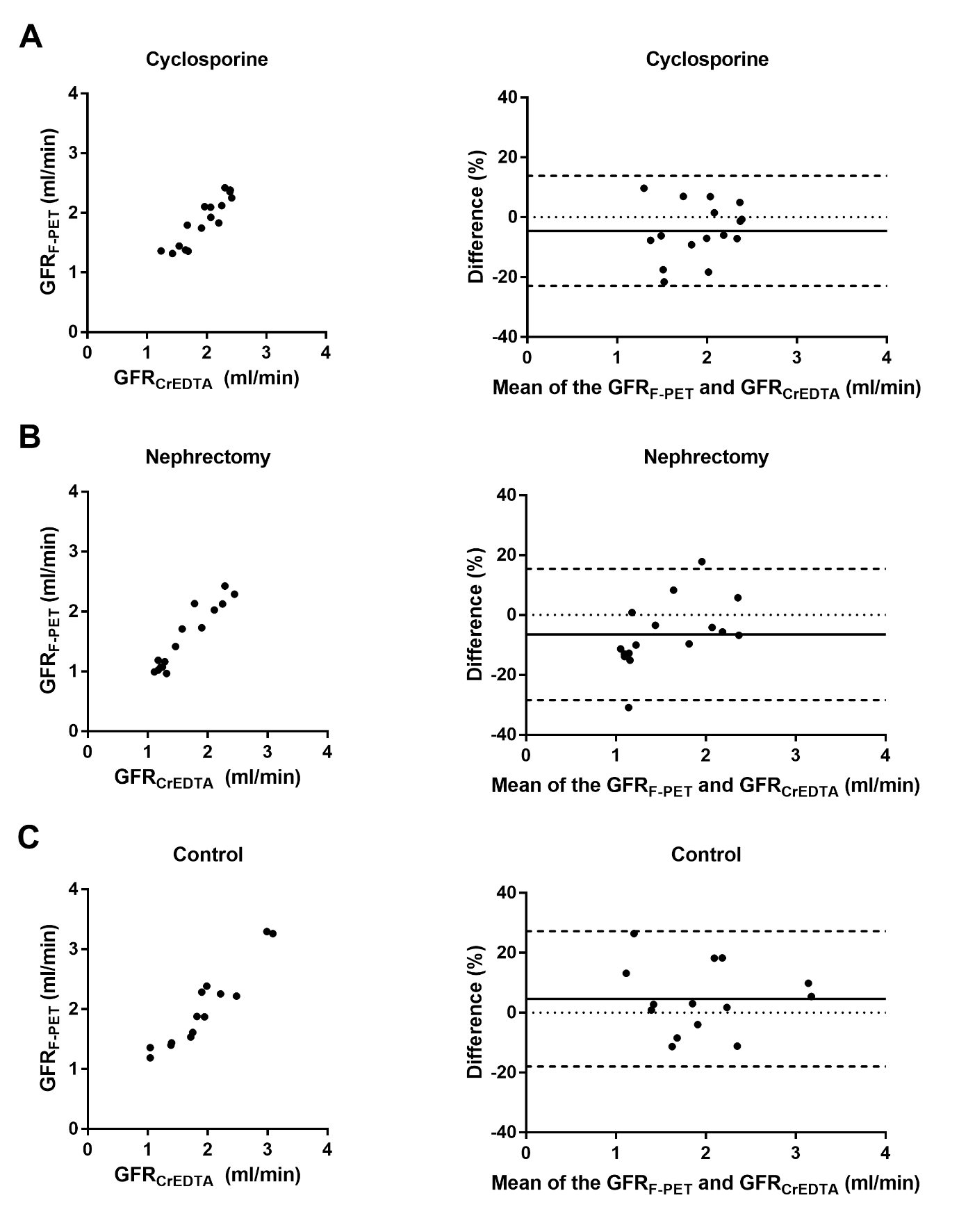


**Supplementary Figure 3.** Agreement between GFR_F-PET_ and GFR_CrEDTA_ in the (A) cyclosporine subgroup, (B) nephrectomy subgroup, and (C) control subgroup. Difference (%) = 100×(GFR_F-PET_ – GFR_CrEDTA_)/(mean of GFR_F-PET_ and GFR_CrEDTA_).


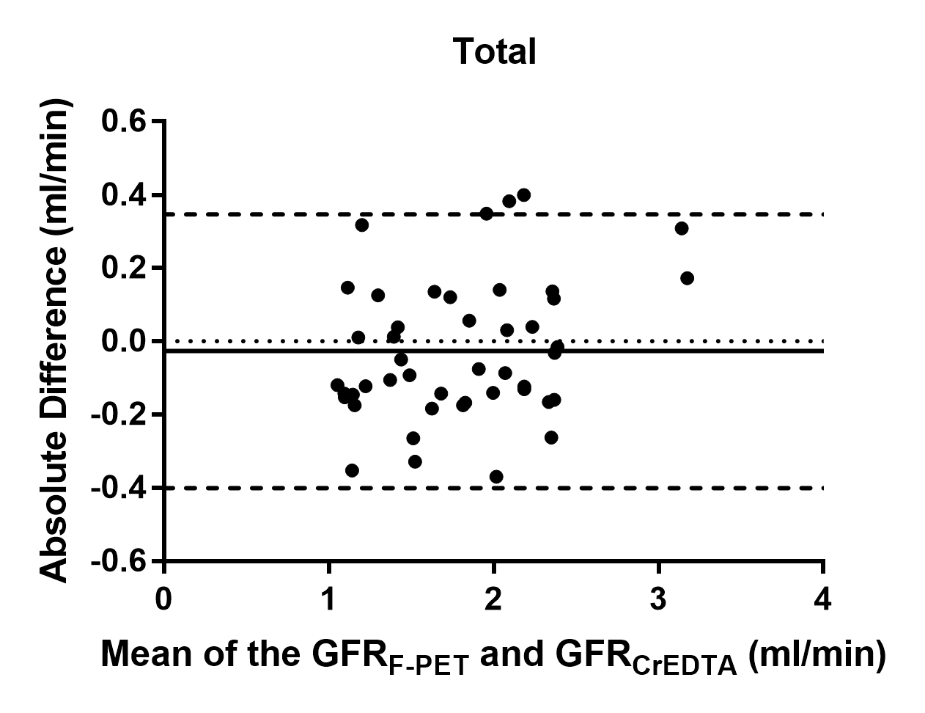


**Supplementary Figure 4.** Bland-Altman plot for absolute differences between GFR_F-PET_ and GFR_CrEDTA_ in the total population (46 measurements). Absolute difference (ml/min) = GFR_F-PET_ – GFR_CrEDTA_


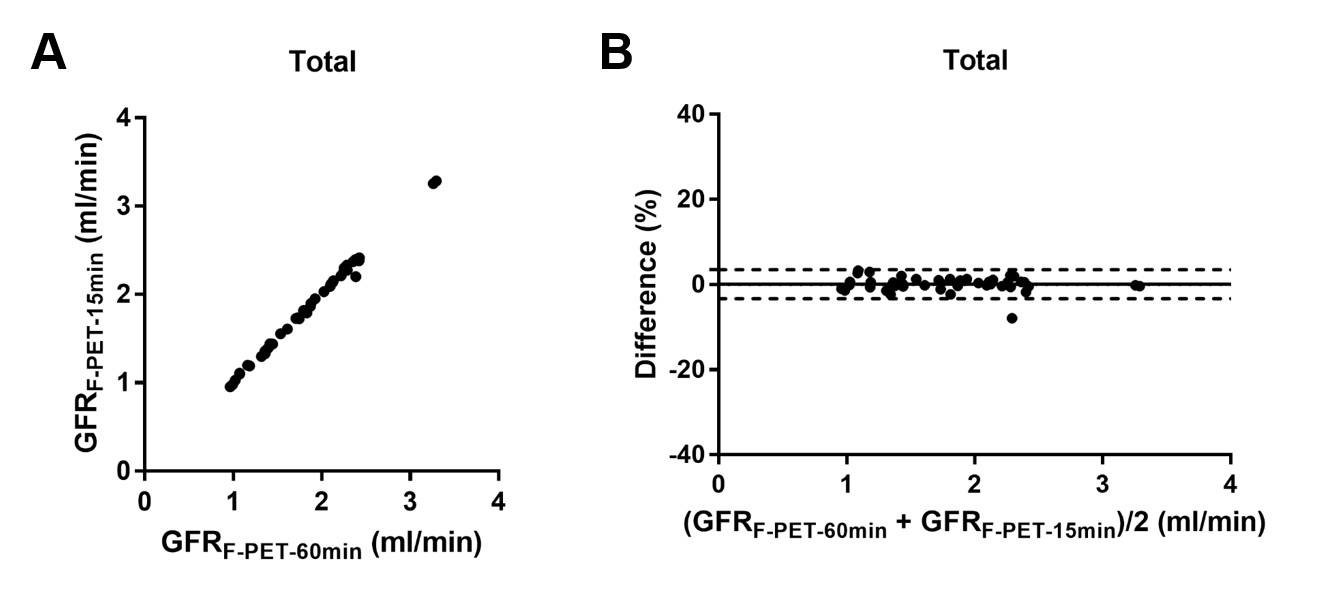


**Supplementary Figure 5.** ^18^F-fluoride PET GFR using 60-and 15-min data. (A) The scatterplot. (B) The Bland-Altman Plot. Difference (%) = 100 × (GFR_F-PET-60min_ – GFR_F-PET-15min_)/(mean of the GFR_F-PET-60min_ and GFR_F-PET-15min_)


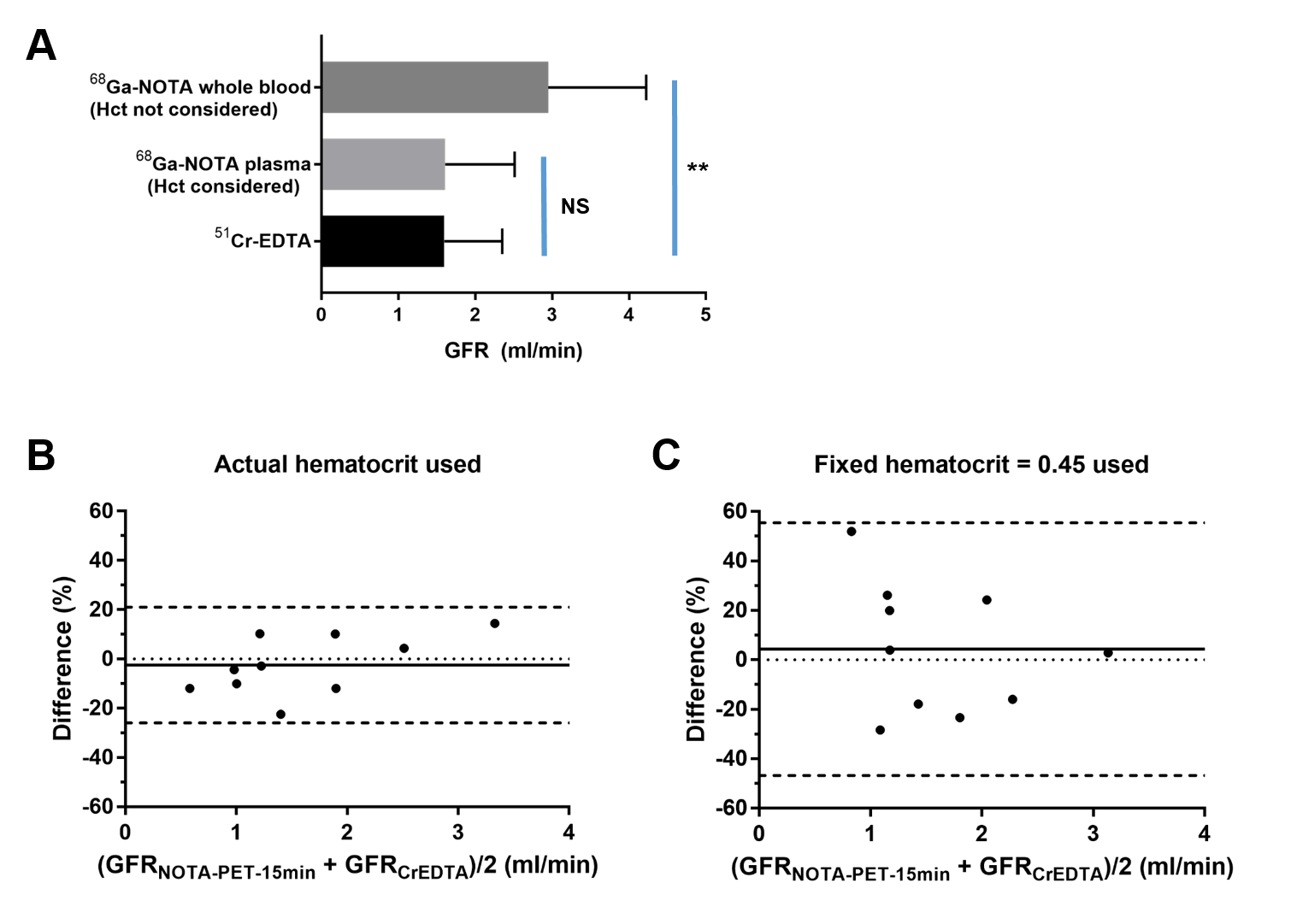


**Supplementary Figure 6.** Requirement of hematocrit measurement for ^68^Ga-NOTA GFR. (A) GFR_NOTA-PET-15min_ produces a significant bias if hematocrit values are not considered in the input function. Hct: hematocrit, NS: non-significant, **: *P* < 0.01. Agreement of GFR_NOTA-PET-15min_ and GFR_CrEDTA_ (B) when measured hematocrit values were used, and **c** when a fixed hematocrit of 0.45 was used. Difference (%) = 100 × (GFR_NOTA-PET-15min_ – GFR_CrEDTA_)/(mean of GFR_NOTA-PET-15min_ and GFR_CrEDTA_)
